# Hydrogen Sulfide Regulates Energy Production to Delay Leaf Senescence Induced by Drought Stress in *Arabidopsis*

**DOI:** 10.3389/fpls.2018.01722

**Published:** 2018-11-23

**Authors:** Zhuping Jin, Limin Sun, Guangdong Yang, Yanxi Pei

**Affiliations:** ^1^School of Life Science, Shanxi University, Taiyuan, China; ^2^Department of Chemistry and Biochemistry, Laurentian University, Sudbury, ON, Canada

**Keywords:** hydrogen sulfide, mitochondrion, ATPase activity, energy production, leaf senescence, drought stress

## Abstract

Hydrogen sulfide (H_2_S) is a novel gasotransmitter in both mammals and plants. H_2_S plays important roles in various plant developmental processes and stress responses. Leaf senescence is the last developmental stage and is a sequential degradation process that eventually leads to leaf death. A mutation of the H_2_S-producing enzyme-encoding gene L-cysteine desulfhydrase1 (*DES1*) leads to premature leaf senescence but the underlying mechanisms are not clear. In this present study, wild-type, *DES1* defective mutant (*des1*) and over-expression (*OE-DES1*) *Arabidopsis* plants were used to investigate the underlying mechanism of H_2_S signaling in energy production and leaf senescence under drought stress. The *des1* mutant was more sensitive to drought stress and displayed accelerated leaf senescence, while the leaves of *OE-DES1* contained adequate chlorophyll levels, accompanied by significantly increased drought resistance. Under drought stress, the expression levels of *ATP*β-*1, -2*, and *-3* were significantly downregulated in *des1* and significantly upregulated in *OE-DES1*, and *ATP*ε showed the opposite trend. Senescence-associated gene (*SAG*) *12* correlated with age-dependent senescence and participated in the drought resistance of *OE-DES1*. *SAG13*, which was induced by environmental factors, responded positively to drought stress in *des1* plants, while there was no significant difference in the *SAG29* expression between *des1* and *OE-DES1*. Using transmission electron microscopy, the mitochondria of *des1* were severely damaged and bubbled in older leaves, while *OE-DES1* had complete mitochondrial structures and a homogeneous matrix. Additionally, mitochondria isolated from *OE-DES1* increased the H_2_S production rate, H_2_S content and ATPase activity level, as well as reduced swelling and lowered the ATP content in contrast with wild-type and *des1* significantly. Therefore, at subcellular levels, H_2_S appeared to determine the ability of mitochondria to regulate energy production and protect against cellular aging, which subsequently delayed leaf senescence under drought-stress conditions in plants.

## Introduction

Hydrogen sulfide (H_2_S) is an important member of the gasotransmitter family, along with nitric oxide and carbon monoxide, in both mammals and plants (Wang, 2012). H_2_S has a wide range of physiological roles that are critical for plant development, such as seed germination (Zhang et al., 2008), root elongation (Zhang et al., 2009), flowering (Zhang et al., 2011), leaf senescence (Álvarez et al., 2010) and fruit maturation (Dooley et al., 2013). H_2_S not only regulates the progress of cell viability (Álvarez et al., 2010), autophagy (Álvarez et al., 2012; Gotor et al., 2013), stomatal movement (Jin and Pei, 2016) and photosynthesis (Chen et al., 2011), but also mediates tolerance and protection against many different environmental stresses, including heat (Li et al., 2012), cold (Du et al., 2017), drought (Jin et al., 2011, 2013, 2017), heavy metals (Fang et al., 2016, 2017), and others (Jin and Pei, 2015). H_2_S is endogenously produced and metabolized in a precise and regulated manner. Cysteine degradation by CDes catalyzes the formation of sulfide, ammonia and pyruvate in a 1:1:1 stoichiometric ratio (Papenbrock et al., 2007). Plant cells contain different CDes localized in the cytoplasm, plastids and mitochondria (Jin and Pei, 2015). One class of these enzymes, L-Cys desulfhydrases, containing LCD (At3g62130) and DES1 (At5g28030), localize in the nucleus and cytoplasm, respectively. DES1 is regarded as the major contributor to the generation of H_2_S (Álvarez et al., 2010).

Leaf senescence, as the last developmental stage, occurs gradually and is characterized by specific macroscopic, cellular, biochemical and molecular changes. Nutrient reallocation is accompanied by organelle breakdown, energy reduction and gene expression during senescence (Munné-Bosch and Alegre, 2004). Mitochondria, the energy powerhouses, play important roles in the cell redox homeostasis, and mitochondrial ATP production is required in both heterotrophic and photosynthetic cells (Noctor et al., 2007). The conversion of the electrochemical proton gradient into ATP is catalyzed by an ATP synthase, often called F_0_F_1_-ATPase, which is located in the inner mitochondrial membrane (Rondelez et al., 2005). ATP synthesis occurs sequentially on the three β-subunits of the F_1_-ATPase (α3β3γδε), while the ε subunit is an endogenous inhibitor of the ATPase activity of F_1_ (Kato et al., 1997). The decreased activity of the F_1_-ATPase may be the main cause of age-dependent mitochondrial dysfunction (Kadoya et al., 2011). Age-related deficits and damage to cellular macromolecules involved in energy production may underlie the age-related lowered ATP production level (Wang et al., 2003). ATP levels drop significantly in stressed cells and increase in the recovered cells (Sõti et al., 2003). Senescence-associated gene (*SAG*) *12*, *13* and *29* are widely used as molecular markers for leaf senescence (Chen et al., 2014).

Mitochondrial morphology and function are preserved by H_2_S when mammals suffer sepsis (Aslami et al., 2013). The endogenous H_2_S remains a regulator of energy production in mammalian cells under stress conditions (Fu et al., 2012) and delays cellular senescence by attenuating oxidative stress (Yang et al., 2013). In *Arabidopsis thaliana*, a mutation of the *DES1* gene leads to early-flowering, premature leaf senescence and cadmium sensitivity (Álvarez et al., 2010). The mRNA levels of H_2_S-encoding genes, L/D-CDes, are gradually elevated in a developmental stage-dependent manner, and the H_2_S production rate is positively correlated with the extent of the drought stress (Jin et al., 2011). H_2_S prolongs the longevity of fresh-cut flowers and kiwifruit, suggesting that the role of H_2_S might be universal in plant senescence (Zhang et al., 2011; Gao et al., 2013). However, the underlying mechanisms of H_2_S signaling in plant senescence and the aging process remain unclear.

Plant aging and environmental stresses may induce the process of senescence. Drought induces several responses in plants, including leaf senescence, which plays a major role in the survival of several species (Munné-Bosch and Alegre, 2004). In the present study, we focused on the role of endogenous H_2_S signals as responses for plant survival and, more specifically, on how these signals contribute to the energy production of mitochondria during leaf senescence under drought-stress conditions.

## Materials and Methods

### Plant Materials and Growth Conditions

*Arabidopsis thaliana* ecotype Columbia*-0* (wild-type, WT) and T-DNA insertion mutant of *DES1* (SALK_205358C) seeds were obtained from the Arabidopsis Biological Resource Center (ABRC^1^). The result of genotyping showed that *des1* was a null mutant (Supplementary Figure S1). Construction of a transformation vector (35S::*DES1*) and transformation of Columbia*-0* by the floral dip method were performed previously. Transformants were selected on 1/2 MS agar plates containing 20 mg L^−1^ hygromycin. The T3 seeds which did not segregate on 1/2 MS plates with hygromycin, were identified as homozygous OE lines. After the comparison of seed germination under PEG-8000 stress and qRT-PCR analysis, one of them was used in the following experiments (Supplementary Figure S2). Seeds of WT, homozygous transgenic (*OE-DES1*) and mutant (*des1*) were vernalized for 2 ∼ 4 days at 4°C and then grown in a nutrient soil: vermiculite (1:1, v/v) mixture in a growth chamber. The growth chamber was maintained at 23 ± 1°C with 60% relative humidity, light illumination of 160 μE⋅m^−2^⋅s^−1^ and a photoperiod of 16/8 h (light/dark).

The 4-week-old WT, *des1* and *OE-DES1* seedlings were subjected to drought stress by withholding water for 7 days, and their growth states were recorded. Each associated gene expression level was determined in WT, *des1* and *OE-DES1*. The leaves of plants were collected, immediately frozen in liquid nitrogen and stored at −80°C.

### Extraction of Total RNA and Quantitative Real-Time PCR (qRT-PCR)

Total RNA was extracted using TRIzol^®^ Reagent (Invitrogen) according to the manufacturer’s instructions. Then, the RNA sample, M-MLV reverse transcriptase and an oligo(dT18) primer (TransGen Biotech) were used to synthesize complementary cDNA, and qRT-PCR was performed using a Bio-Rad real-time PCR Detection System (in a CFX96TM C1000 thermal cycler). In the relative quantification analysis, the gene *UBQ4* was used as the internal control. Each experiment was performed in triplicate and repeated independently with three biological replicates (Table 1).

**Table 1:** List of primers for qRT-PCR.

Gene	Accession number	Primer pairs
*ATP*β-*1*	At5g08670	5′-ACGGACAAATGAATGAGCC-3′
		5′-ACAGCAGACGGGATACGAC-3′
*ATP*β-*2*	At5g08690	5′-TGTGGCTGAGTATTTCCGTGAT-3′
		5′-GCAGGGACATAGATGGCTTG-3′
*ATP*β-*3*	At5g08680	5′-GACAACATCTTCCGTTTCAC-3′
		5′-ATGGCTTGGACAGAGGTAAT-3′
*ATP*ε	At1g51650	5′-ATGCGGCGGTTCCGTTCT-3′
		5′-AGGTTTCTGGGGCTTTCC-3′
*SAG12*	At5g45890	5′-ACTGGTTTCAAAGGTGTCTCGGCAT-3′
		5′-ACGCCCAACAACATCCGCAGC-3′
*SAG13*	At2g29350	5′-AGCGACAACATAAGGACGAAC-3′
		5′-AGACAAAGAAATGCCACAAGC-3′
*SAG29*	At5g13170	5′-TCGGCATCTTAGGAAACG-3′
		5′-CGGTAGCGACTGGAAACT-3′
*UBQ4*	At5g20620	5′-GGGCACTCAAGTATCTTGTTAGC-3′
		5′-TGCTGCCCAACATCAGGTT-3′

### Observation of Mitochondrial Ultrastructure Using Transmission Electron Microscopy (TEM)

To analyze the ultrastructure of mitochondria by TEM, 2-, 4-, 6-, and 8-week-old plants were subjected to drought stress and cultured in pots without water for 1 week. Leaves were collected and fixed for 24 h in 2.5% glutaraldehyde in 0.1 M phosphate buffer (pH 7.2) at 4°C, and then thoroughly washed with the same buffer three times (10 min per wash). Subsequently, samples were post-fixed in 2% osmium tetroxide in the same buffer for 4 h. Samples were dehydrated gradually by increasing concentrations of ethyl alcohol (25, 50, 75, 85, 95, and 100%) and embedded in Epon 812 resin. Following overnight embedding and the polymerization of the embedding medium, ultrathin sections were cut using an ultramicrotome (LACA-UC6) with a diamond knife (70 nm). Finally, samples were stained with uranyl acetate and lead citrate. They were then examined using a transmission electron microscope (JEOL, JEM-1400) and photographed.

### Isolation and Purification of Mitochondria From Leaves

The intact mitochondria were isolated and purified following a protocol described previously, with some modifications (Rödiger et al., 2010). Leaves of 4-week-old were collected and then exact 5 g of each sample were ground in 15 mL of isolation buffer, containing 1 mM DL-dithiothreitol, 1% (w/v) polyvinylpyrrolidone-40 and 1% (w/v) defatted bovine serum albumin, adjusted to pH 7.5 with KOH. The extraction was filtered through 2 layers of 25-μm nylon mesh and centrifuged for 10 min at 3000 *g*. The supernatant was transferred to a new tube and centrifuged for 15 min at 20000 *g*, and the sediment was resuspended in 15 mL washing buffer before repeating the above steps. The resulting supernatant was discarded and the pellet was resuspended in 1 mL of washing buffer. The pellet’s supernatant was carefully layered on top of a Percoll gradient and centrifuged for 60 min at 40000 *g*. The mitochondrial fraction, which accumulated at the interphase between 27 and 50% Percoll, was collected. The sediment was diluted with 1 mL of washing buffer and centrifuged for 15 min at 20000 *g*. This was repeated three times. Finally, the pellet was resuspended in 1 mL of preservation buffer and stored. All steps involved in isolating mitochondria were conducted at 4°C. Janus green B which has been considered specific for mitochondria was used to promise the isolated mitochondria were viable (Supplementary Figure S3) (Lyons et al., 1964).

### Measurement of Mitochondrial Activity and Mitochondrial Swelling

The mitochondrial activity was determined according to previously described methods with some modifications (Du et al., 2014). Take 100 μL of freshly prepared mitochondrial suspension sample stored at 4°C (with 100 μL suspension buffer as blank control), add 40 μL of 5 mg⋅mL^−1^ MTT (3-(4,5-Dimethylthiazol-2-yl)-2,5-Diphenyltetrazolium Bromide), incubate at 30°C for 30 min, then add 100 μL of isopropanol at room temperature for 20 min, and the absorbance value of A_570_ was measured using a multi-function microplate reader, and the value thereof reflected mitochondrial activity.

Freshly prepared mitochondria were kept at 4°C prior to the reaction. The mitochondria removed rapidly in to the medium (25 mM sucrose, 0.5 mM KH_2_PO_4_, 1 mM Sodium succinate, and pH 7.2). Mitochondrial swelling was determined in all groups by measuring the change in the absorbance of the mitochondrial suspension at 540 nm. The reaction conditions were set at 25°C. According to this method, the more serious the mitochondrial swelling is, the smaller absorbance value at 540 nm becomes (Du et al., 2014).

### Measurement of ATPase Activity and ATP Content in Mitochondria

ATPase activity was assayed by measuring inorganic phosphate (Pi) from ATP, and Pi was determined according to Ames (Toribarn et al., 1966). In brief, ATPase activity was measured in 0.5 mL of reaction solution contained 0.25 mL mitochondrial suspension, 3 mM ATP, 50 mM KCl, 1.5 mM MgCl_2_, 50 mM NaCl and 33 mM Tris titrated to pH 6.5 with HCl. Corrections were made using blanks without ATP. After the incubation of 30 min at 38°C, 100 μL 20% (w/v) trichloroacetic acid quenched the reaction. The liberated Pi was measured with a spectrophotometer at 700 nm. ATPase activity was expressed in μmol Pi per mg protein per min, and the protein content was determined by the method (Lowry et al., 1951).

The amount of ATP in mitochondria was measured using the luciferin-luciferase assay. The assay was analyzed with an ENLITEN ATP assay bioluminescence detection kit, according to the manufacturer’s recommended protocol. In addition, operational steps also based on previously published methods were performed with minor modification (Goh et al., 2004). The luminescence was integrated for 10 s using a multifunctional microplate reader. The actual ATP levels were calculated from an ATP standard curve, which was constructed using different concentrations of commercially supplied ATP.

### Measurement of the Cys Content in Mitochondria

The L-Cys contents of mitochondria were quantitated using a method described previously (Fu et al., 2012). Briefly, 100 μL samples were prepared from mitochondrial suspensions. L-Cys reacts with glacial acetic acid and ninhydrin reagent specifically, which has a maximum absorbance at 560 nm.

### Measurement of the H_2_S Production Rate and Content in Mitochondria

Fresh mitochondrial suspensions were prepared to determine the production rate of H_2_S from L-Cys and D-Cys. The assay was performed in accordance with methods described previously (Fu et al., 2012).

The endogenous H_2_S contents of mitochondria were determined following the protocol described previously (Jin et al., 2017). Freshly prepared mitochondrial suspensions were homogenized with 1.5 mL of extraction buffer. The samples were then analyzed using a Four-channel Free Radical Analyzer with tissue electrodes. Different concentrations of NaHS (donor of H_2_S) were used against the available standard H_2_S measurement curve.

### Statistical Analyses

Each experiment was performed in triplicate, each with at least three biological replicates. All data were presented as the means ± SEs. The results were statistically analyzed using a one-way analysis of variance with SPSS 16.0 software, and error bars were determined based on Tukey’s multiple range test. *P*-value < 0.05 was considered statistically significant.

## Results

### The H_2_S Content Is Correlated With Leaf Senescence During Drought Stress

**FIGURE 1:**
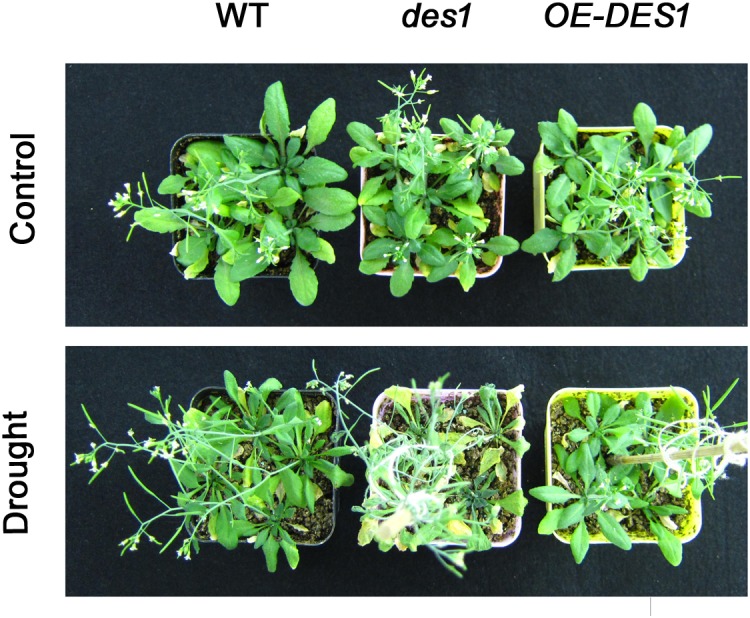
Effects of endogenous H_2_S on seedling growth. The 4-week-old WT, *des1* and *OE-DES1* seedlings were subjected to drought stress by withholding water for 7 days, and then the growth states of seedlings were recorded.

The H_2_S contents in leaves from different plants were quantified. Compared with WT, the H_2_S content of the *des1* mutant decreased significantly and that of *OE-DES1* increased significantly (Jin et al., 2017). The effects of H_2_S on seedling growth under normal and drought-stress conditions were observed. Under normal conditions, the development of *des1* was much slower than WT and *OE-DES1*. Most leaves of *des1* showed wilting and turned yellow after 5 days under drought-stress conditions, while leaves of WT curled severely and those of *OE-DES1* were green and expanded (Figure 1).

### Endogenous H_2_S Affects the Transcriptional Levels of *SAGs* and ATP Synthase-Related Genes

**FIGURE 2:**
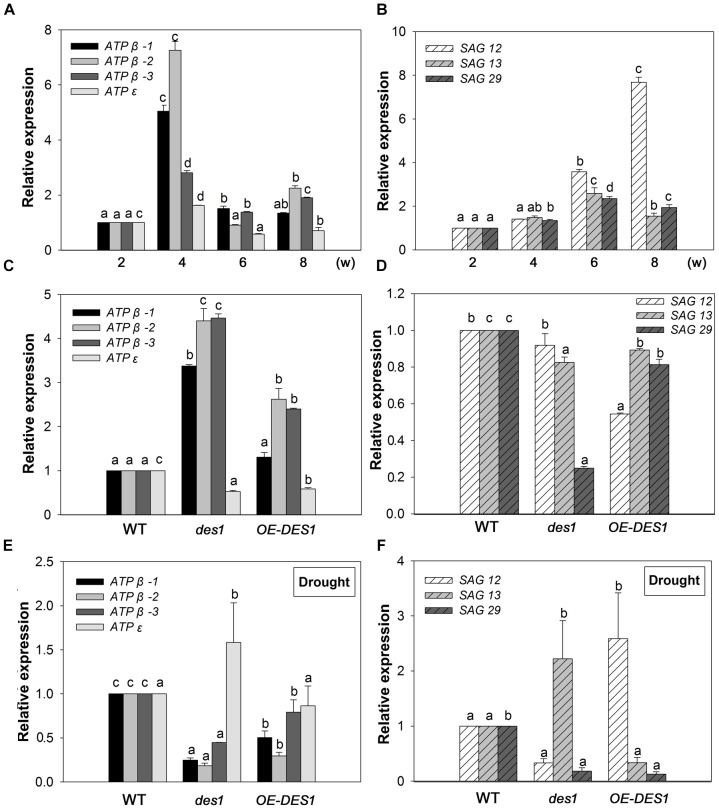
Expression patterns of *SAG*s and ATP synthase-related genes. **(A)** Expression patterns of ATP synthase-related genes at different developmental stages. **(B)** Expression patterns of *SAG*s at different developmental stages. **(C)** Effects of endogenous H_2_S on the expression levels of ATP synthase-related genes. **(D)** Effects of endogenous H_2_S on the expression levels of *SAG*s. **(E)** Expression levels of ATP synthase-related genes under drought-stress conditions in WT, *des1* and *OE-DES1*. **(F)** Expression levels of *SAG*s under drought-stress conditions in WT, *des1* and *OE-DES1*. Data are means ± SEs of three independent experiments; bars marked with the same letter did not differ significantly at *p* < 0.05.

The gene expression levels of *ATP*β-*1*, *ATP*β-*2*, *ATPβ-3*, *ATP*ε, *SAG12*, *SAG13*, and *SAG29* at the different growth stages of WT plants were analyzed using qRT-PCR with mRNA from the leaves of 2-, 4-, 6-, and 8-week-old plants. Compared with the expression at 2 weeks, the levels of *ATP*β-*1∼3* and *ATP*ε were stimulated by 5-, 7.25-, 2.8-, and 1.6-fold at 4 weeks, respectively, and then dropped at 6 and 8 weeks (Figure 2A). During this period, the expression levels of *SAG12*, *13* and *29* gradually increased. *SAG12* was the greatest responder, with expression up-regulated by 3.58-fold at 6 weeks and 7.67-fold at 8 weeks compared with the levels at 2 weeks (Figure 2B).

To study the effects of drought stress on the expression levels of these genes, qRT-PCR was performed using mRNA from the leaves of plants grown under two different conditions. Under normal conditions, the expression levels of *ATP*βs increased significantly in *des1* and *OE-DES1* compared with WT, while *ATP*ε decreased significantly (Figure 2C). The expression level of *SAG12* was not different between *des1* and WT, but decreased significantly in *OE-DES1*. Additionally, *SAG13* and *SAG2*9 expression levels were significantly down-regulated in *des1* and *OE-DES1* compared with WT (Figure 2D). Under drought-stress conditions, the expression levels of *ATP*βs were significantly lower in *des1* and *OE-DES1*, and that of *ATP*ε was greater in *des1* compared with WT (Figure 2E). At the same time, compared with WT, *SAG12* expression reduced in *des1* but significantly enhanced in *OE-DES1*, while *SAG13* had the opposite trend. *SAG29* expression was significantly down-regulated in both *des1* and *OE-DES1* (Figure 2F).

### Endogenous H_2_S Protected the Mitochondrial Ultrastructure in Leaves Under Drought-Stress Conditions

**FIGURE 3:**
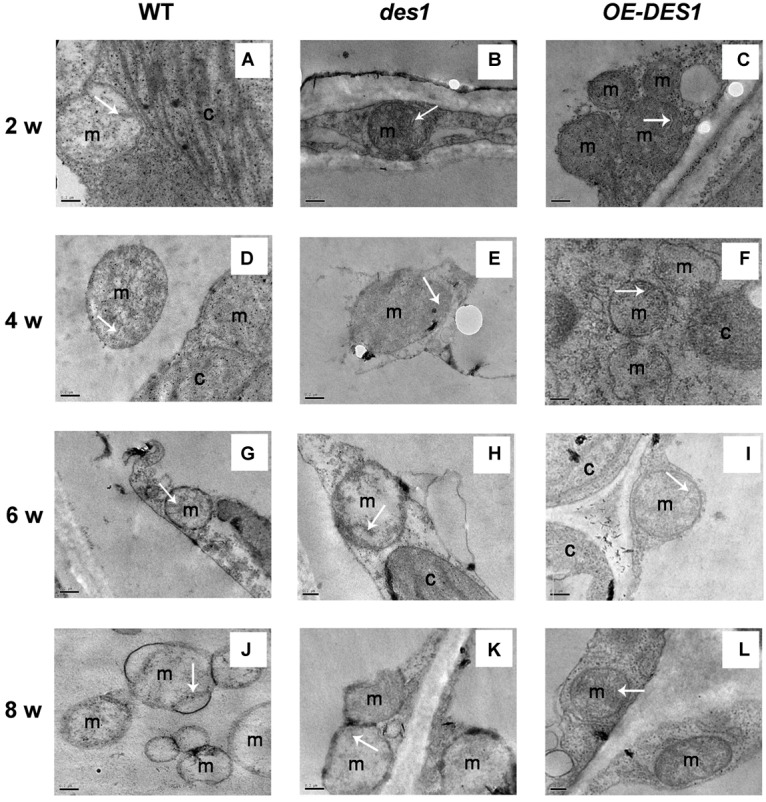
Effects of H_2_S on mitochondrial ultrastructure in WT, *des1* and *OE-DES1* plants at different developmental stages under drought-stress conditions. The 1-, 3-, 5-, and 7-week-old seedlings were subjected to drought stress by withholding water for 7 days, and then samples of leaves were collected. Ultrastructure of WT **(A,D,G,J)**, *des1* **(B,E,H,K)**, and *OE-DES1* **(C,F,I,L)** were observed using TEM. Bars = 0.2 μm. M, mitochondrion; C, chloroplast. The tubular shape or remnants of cristae were labeled by arrows.

Leaves of plants at different developmental stages were collected after withholding water for 1 week, and the ultrastructure of the mitochondria were observed using TEM. The representative ultrastructures at lower magnification are shown as Supplementary Figure S5. In 2-week-old young rosette tissues, the mitochondria had intact membranes, elaborate cristae and homogeneous matrixes were seen in all of the materials (Figures 3A–C). With seedling development, the mitochondrial membrane became deformed and the cristae swelled gradually in WT and *des1*, while *OE-DES1* seedlings displayed relatively normal and numerous mitochondria (Figures 3D–I). In the aging leaves under drought stress for 1 week, the mitochondria of *des1* lost their internal structures and swollen cristae, which had internal contents that degraded and membrane that became incomplete. WT samples followed a similar change and the mitochondrial cristae suffered serious damage and tended to bubble. The mitochondrial structures of *OE-DES1* were intact, with mitochondrial cristae having a tubular shape and no obvious swelling (Figures 3J–L).

### Endogenous H_2_S Affected Mitochondrial Activity, Mitochondrial Swelling, ATPase Activity and ATP Content

**FIGURE 4:**
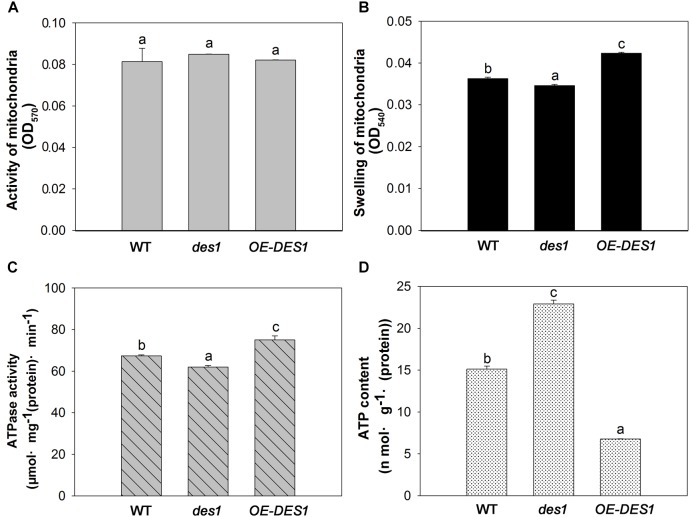
Effects of H_2_S on mitochondrial functions in WT, *des1* and *OE-DES1* under drought-stress conditions. Leaves of 4-week-old after withholding water for 1 week were collected and the mitochondria were isolated and purified. **(A)** Activity levels of mitochondria and **(B)** swelling of mitochondria according to the method previously described (Du et al., 2014). **(C)** ATPase activity levels of mitochondria according to the method previously described (Toribarn et al., 1966). **(D)** ATP contents of mitochondria in WT, *des1* and *OE-DES1* were measured by the luciferin-luciferase (Promega, Madison, WI, United States). Data are means ± SEs of three independent experiments; bars marked with the same letter did not differ significantly at *p* < 0.05.

To identify the effects of H_2_S on the physiological functions of mitochondria, the mitochondrial fractions in WT, *des1* and *OE-DES1* were independently isolated. The viability of mitochondria was detected with Janus Green B (Supplementary Figure S3). To control the purity of our mitochondrial fractions, the expressions of the marker protein in chloroplast and mitochondrion were determined, respectively (Supplementary Figure S4). Under drought-stress treatments, the mitochondrial activity was assessed using an MTT colorimetric assay at OD_570_. No significant differences were found among WT, *des1* and *OE-DES1* (Figure 4A). The swelling of mitochondria is closely related to their activity levels. Consistent with the ultrastructure results, the swelling of mitochondria significantly increased in *des1* and significantly decreased in *OE-DES1* compared with WT (Figure 4B). To further investigate the function of H_2_S on energy production in mitochondria, the ATPase activity levels and ATP contents of mitochondria were detected. Under drought-stress conditions, ATPase activity levels of mitochondria decreased significantly in *des1* and increased significantly in *OE-DES1* compared with the WT (Figure 4C). In contrast, an opposite trend occurred for the ATP contents of different mitochondria, which indicated that *des1* has significantly more ATP and *OE-DES1* has significantly less in comparison with WT (Figure 4D).

### Relationships Among the Cys Content, H_2_S Content and H_2_S Production Rate in Mitochondria

**FIGURE 5:**
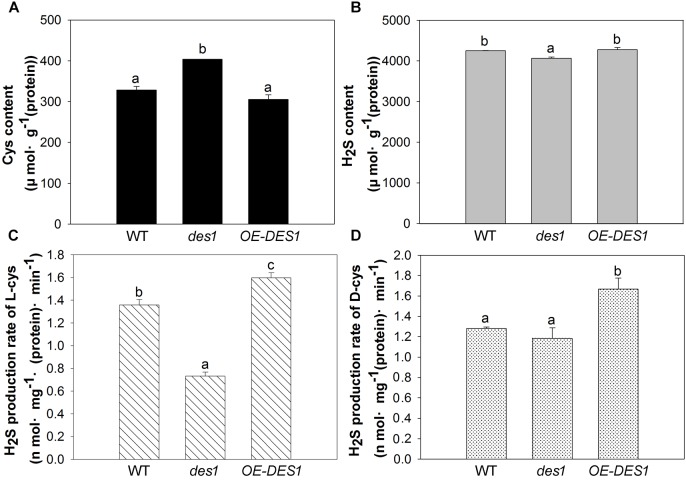
Effects of drought stress on the H_2_S-related physiological indices in the mitochondria of WT, *des1* and *OE-DES1*. Leaves of 4-week-old after withholding water for 1 week were collected and the mitochondria were isolated and purified. **(A)** Cys contents of mitochondria were quantitated by the method described previously (Fu et al., 2012). **(B)** H_2_S contents of mitochondria were measured with electrodes (TBR4100 WPI, Sarasota, FL, United States). **(C)** H_2_S production rates of L-Cys and **(D)** H_2_S production rates of D-Cys in mitochondria of WT, *des1* and *OE-DES1* were measured with methylene blue method (Fu et al., 2012). Data are means ± SEs of three independent experiments; bars marked with the same letter did not differ significantly at *p* < 0.05.

To further confirm the regulatory role of H_2_S on ATP production in mitochondria, we isolated the mitochondrial fractions and detected the contents of Cys and H_2_S, along with the H_2_S production rate, in different cysteine substrates. Drought stress induced a significant increase in the Cys content of *des1* compared with WT, and there was no significant difference in the content between *OE-DES1* and WT (Figure 5A). The H_2_S content revealed opposite results (Figure 5B). The enzyme-catalyzed degradation of Cys is considered the main pathway of H_2_S production in plants. As expected, regardless of whether there was the L-Cys or D-Cys substrate, the production rate of H_2_S increased significantly in *OE-DES1* and decreased significantly in *des1*, producing comparable yields (Figures 5C,D).

## Discussion

Leaf senescence represents a key developmental phase in the lives of both annual and perennial plants, and is as ordered and complex as any other developmental phase (Buchanan-Wollaston, 1997). A large range of cDNA clones, representing genes, have been isolated and showed increased expression levels in senescing leaves. In our studies, the elevated expression of *SAG12* is closely correlated with age-dependent senescence (Figure 2B), which was in agreement with a previous report (Kim et al., 2008), and revealed that senescence began with 6-week-old leaves. Furthermore, the expression level of *SAG12* decreased significantly in *OE-DES1* under normal growth conditions compared with in WT (Figure 2D). In contrast, expression of *SAG13* gradually increased with development and was significantly greater in *des1* than in WT and *OE-DES1* under drought-stress conditions (Figure 2F), which was specifically induced by environmental factors (Meyer et al., 2003). *SAG29* is expressed primarily in senescing plant tissues and serves as a molecular link that integrates environmental stress responses into the senescence process (Chen et al., 2014). As shown here, the expression of *SAG29* was significantly greater in *OE-DES1* than in *des1* under normal growth conditions (Figure 2D) and was not significantly different between *des1* and *OE-DES1* under drought-stress conditions (Figure 2F). Therefore, endogenous H_2_S may regulate leaf senescence by participating in *SAG*s’ expression under normal and drought-stress conditions.

Leaf senescence involves a genetically programmed loss of metabolic function associated with programmed cell death as the terminal phase. Mitochondria, which have elaborate cristae in young and mature *Arabidopsis* rosette tissues, only lose their internal structure, with swollen cristae, at the final programmed cell death stage of senescence when most of the cellular proteins and other reserves have been degraded and exported (Noctor et al., 2007). Thus, we observed the ultrastructures of mitochondria in leaves having unequal H_2_S contents. Although the cristae in all cells swelled gradually during development, differences among the three plant materials from the same period were apparent. Mitochondrial damage was slightly mitigated in *OE-DES1* compared with in the WT and *des1* under drought-stress conditions (Figure 3). The absorbance at OD_540_, representing swelling of mitochondria, corroborated the previous result (Figure 4B). In consequence, physiological H_2_S may protect the mitochondrial structures from injury induced by drought stress during plant senescence, which is consistent with previous studies in animals (Aslami et al., 2013; Yang et al., 2013; Du et al., 2014).

The three β subunits, having an alternate arrangement in the ATPase complex, catalyze the synthesis of ATP from ADP and Pi, and the ε subunit is an endogenous inhibitor of the ATPase activity that may regulate ATPase activity (Kadoya et al., 2011). In the present study, the expression levels of *ATP*βs and *ATP*ε were significantly up-regulated by 4 weeks and then declined (Figure 2A), which might indicate that the demand for ATPase was greater at the seeding stage but lower during leaf senescence. *ATP*βs’ expression levels significantly increased in *des1* under normal conditions but decreased under drought-stress conditions compared with *OE-DES1*, while the expression of *ATP*ε showed the opposite trend during the same time (Figures 2C,E). Under drought-stress treatment, the ATPase activities of mitochondria markedly increased in *OE-DES1* and decreased in *des1* compared with WT (Figure 4C). The ATPase activity was in accordance with the expression levels of ATPase genes (Figures 2E, 4C), suggesting that H_2_S enhanced the activity of ATPase by upregulating the expression levels of *ATP*βs and downregulating the expression level of *ATP*ε in response to leaf senescence induced by drought stress.

High ATP synthase activity means high ATP production. However, in contrast to the ATPase activity, the ATP concentration was the greatest in *des1* and the lowest in *OE-DES1* compared with WT (Figures 4C,D). In other words, there is a lower ATPase activity which may corroborate with an accumulation of ATP in *des1*, and vice versa in *OE-DES1*. In fact, the ATP content we measured shows the sum of positive ATP generation and negative ATP consumption. That is, ATP consumption might be higher in *OE-DES1* which has lower ATP content. In addition, it is also plausible that the lower H_2_S content measured in *des1* impacts the ATP production under drought-stress conditions. It has previously been reported in mammals that H_2_S decreases the ATP content of mitochondria in smooth muscle cells under hypoxia-stress conditions and in brain cells under normoxic conditions (Fu et al., 2012). Individuals with high metabolic capacities may possess adaptations against aging (Niitepõld and Hanski, 2013). Thus, we hypothesized that H_2_S might preserve ATPase activities to meet the needs of the higher metabolism in *OE-DES1*, which had leaves that were relatively younger during drought stress (Figure 1).

DES1, localized in the cytoplasm, is the main enzyme producing H_2_S in plants. So, it is very interesting that *DES1* mutation affected the H_2_S production in mitochondrion. Why? It has been reported that cystathionine γ-lyase (the main enzyme of H_2_S production in animal cells, also localized in cytoplasm) can translocate to mitochondria on hypoxic stress stimulation. Subsequently H_2_S production inside mitochondria was promoted, and mitochondrial ATP production was sustained (Fu et al., 2012). As eukaryotes, there are many similarities in their physiological and biochemical processes in both animals and plants. Especially in the field of gasotransmitter research, numerous studies have confirmed that the analogy from animal to plant was correct, which provided us with a valuable research idea. It is quite possible that DES1 affects the production of H_2_S in mitochondria and subsequently affects energy production in mitochondria through the similar mechanism of action. It should be actually investigated more deeply in the future.

H_2_S metabolism occurs in mitochondria, and H_2_S improves mitochondrial ATP production in mammalian cells under stress conditions (Fu et al., 2012). The *des1* mutant of *Arabidopsis* displayed premature aging under Cd stress (Álvarez et al., 2010), and H_2_S-induced stomatal closure requires ABC transporter-dependent extracellular ATP production (Wang et al., 2015). In this present study, we explored the regulation of energy produced by endogenous H_2_S in plants. The H_2_S content and H_2_S production rate based on L-Cys and D-Cys were enhanced in the mitochondria of *OE-DES1*, indicating that the above results are effects of H_2_S in mitochondria (Figure 5). In fact, several enzymes related to H_2_S production are localized on the mitochondria, including D-Cys desulphydrase1 (DCD1, At1g48420), DCD2 (At3g26115), cysteine desulfurase (NFS1, At5g65720), cyanoalanine synthase (CYS-C1, At3g61440) and *O*-acetyl-L-serine(thiol)lyase (OASTL-C, At3g59760). We compared their expression levels in WT between normal and drought-stress conditions. They all responded to drought stress. The expression levels of *DCD2*, *CYS-C1*, and *OASTL-C* were significantly increased compared with WT (Supplementary Figure S6), which implies these three proteins may play roles in the regulation of mitochondrial energy metabolism during leaf senescence. Subsequent studies with mutants of these enzymes should clarify the H_2_S-associated regulatory mechanism of mitochondrial energy metabolism.

## Conclusion

**FIGURE 6:**
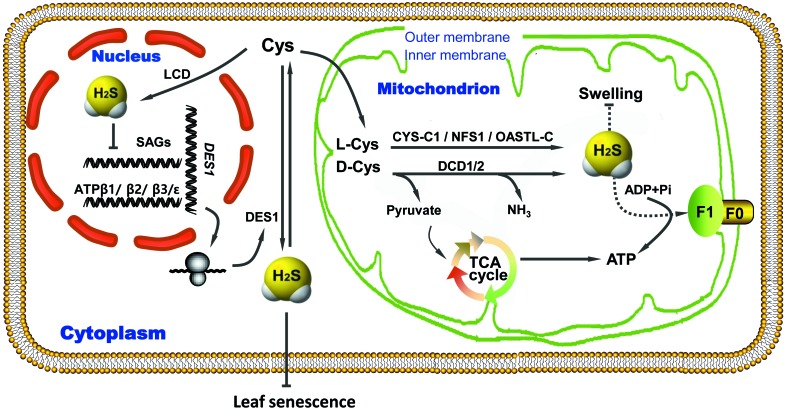
Proposed regulatory model of ATP production induced by endogenous H_2_S to delay leaf senescence under drought-stress conditions. ADP, adenosine diphosphate; ATP, adenosine tiphosphate; Cys, cysteine; CYS, cyanoalanine synthase; DES1, cysteine desulfhydrase; F_0_/F_1_, complex of ATP synthase; H_2_S, hydrogen sulfide; LCD/DCD, L/D-cysteine desulfhydrase; NFS1, cysteine desulfurase; OASTL, *O*-acetyl-L-serine(thiol)lyase; SAGs, senescence-associated genes; TCA, tricarboxylic acid; Arrow end, activation; Blunt end, inactivation.

Endogenous H_2_S mitigates mitochondrial swelling, preserves energy production and protects cellular metabolism during leaf senescence induced by drought stress (Figure 6). As such, H_2_S delays leaf senescence by acting as a regulator of energy production in mitochondria. These findings increase our understanding of the physiological functions of H_2_S and the regulation of energy metabolism in plants.

## Author Contributions

GY and YP designed the original research project. LS and ZJ performed all the experiments. LS and ZJ analyzed the data and wrote the manuscript. GY and YP revised the writing. All authors have read and approved the manuscript.

## Conflict of Interest Statement

The authors declare that the research was conducted in the absence of any commercial or financial relationships that could be construed as a potential conflict of interest.

## Footnotes

1 http://www.arabidopsis.org/abrc/

## Abbreviations

ADP: adenosine diphosphate
ATP: adenosine triphosphate
CYS: cyanoalanine synthase
Cys: cysteine
DES1: cysteine desulfhydrase
*F*_0_/*F*_1_: complex of ATP synthase
H_2_S: hydrogen sulfide
LCD/DCD: L/D-cysteine desulfhydrase
NFS1: cysteine desulfurase
OASTL: *O*-acetyl-L-serine(thiol)lyase
SAGs: senescence associated genes
TCA: tricarboxylic acid.

## References

[B1] Álvarez C. Calo L. Romero L. C. Garcıá I. Gotor C. (2010). An O-acetylserine(thiol)lyase homolog with L-cysteine desulfhydrase activity regulates cysteine homeostasis in Arabidopsis. *Plant Physiol.* 152 656–669. 10.1104/pp.109.147975 19955263 PMC2815857

[B2] Álvarez C. García I. Moreno I. Pérez-Pérez M. E. Crespo J. L. Romero L. C. (2012). Cysteine-generated sulfide in the cytosol negatively regulates autophagy and modulates the transcriptional profile in Arabidopsis. *Plant Cell* 24 4621–4634. 10.1105/tpc.112.105403 23144183 PMC3531856

[B3] Aslami H. Pulskens W. P. Kuipers M. T. Bos A. P. Kuilenburg A. B. P. Wanders R. J. A. (2013). Hydrogen sulfide donor NaHS reduces organ injury in a rat model of pneumococcal pneumosepsis, associated with improved bio-energetic status. *PLoS One* 8:e63497. 10.1371/journal.pone.0063497 23717435 PMC3662774

[B4] Buchanan-Wollaston V. (1997). The molecular biology of leaf senescence. *J. Exp. Bot.* 48 181–199. 10.1093/jxb/48.2.181

[B5] Chen J. Wu F. H. Wang W. H. Zheng C. J. Lin G. H. Dong X. J. (2011). Hydrogen sulphide enhances photosynthesis through promoting chloroplast biogenesis, photosynthetic enzyme expression, and thiol redox modification in Spinacia oleracea seedlings. *J. Exp. Bot.* 62 4481–4493. 10.1093/jxb/err145 21624977 PMC3170546

[B6] Chen M. Maodzeka A. Zhou L. Ali E. Wang Z. Jiang L. X. (2014). Removal of DELLA repression promotes leaf senescence in Arabidopsis. *Plant Sci.* 21 26–34. 10.1016/j.plantsci.2013.11.016 24576761

[B7] Dooley F. D. Nair S. P. Ward P. D. (2013). Increased growth and germination success in plants following hydrogen sulfide administration. *PLoS One* 8:e62048. 10.1371/journal.pone.0062048 23614010 PMC3629089

[B8] Du Q. Wang C. Zhang N. Li G. F. Zhang M. Li L. P. (2014). In vivo study of the effects of exogenous hydrogen sulfide on lung mitochondria in acute lung injury in rats. *BMC Anesthesiol.* 14:117. 10.1186/1471-2253-14-117 25550681 PMC4279795

[B9] Du X. Z. Jin Z. P. Liu D. M. Yang G. D. Pei Y. X. (2017). Hydrogen sulfide alleviates the cold stress through MPK4 in *Arabidopsis thaliana*. *Plant Physiol. Biochem.* 120 112–119. 10.1016/j.plaphy.2017.09.028 29024849

[B10] Fang H. H. Liu Z. Q. Jin Z. Zhang L. P. Liu D. M. Pei Y. X. (2016). An emphasis of hydrogen sulfide-cysteine cycle on enhancing the tolerance to chromium stress in Arabidopsis. *Environ. Pollut.* 213 870–877. 10.1016/j.envpol.2016.03.035 27038574

[B11] Fang H. H. Liu Z. Q. Long Y. P. Liang Y. L. Jin Z. P. Zhang L. P. (2017). The Ca^2+^/calmodulin2-binding transcription factor TGA3 elevates *LCD* expression and H_2_S production to bolster Cr^6+^ tolerance in Arabidopsis. *Plant J.* 91 1038–1050. 10.1111/tpj.13627 28670772

[B12] Fu M. Zhang W. H. Wu L. Y. Yang G. D. Li H. Z. Wang R. (2012). Hydrogen sulfide (H_2_S) metabolism in mitochondria and its regulatory role in energy production. *PNAS.* 109 2943–2948. 10.1073/pnas.1115634109 22323590 PMC3287003

[B13] Gao S. P. Hu K. D. Hu L. Y. Li Y. H. Han Y. Wang H. L. (2013). Hydrogen sulfide delays postharvest senescence and plays an antioxidative role in fresh-cut Kiwifruit. *Hortscience* 48 1385–1392.

[B14] Goh C. H. Jung K. H. Roberts S. K. McAinsh M. R. Hetherington A. M. Park Y. (2004). Mitochondria provide the main source of cytosolic ATP for activation of outward-rectifying K^+^ channels in mesophyll protoplast of chlorophyll-deficient mutant rice (OsCHLH) seedlings. *J. Biol. Chem.* 279 6874–6882. 10.1074/jbc.M309071200 14660680

[B15] Gotor C. García I. Crespo J. L. Romero L. C. (2013). Sulfide as a signaling molecule in autophagy. *Autophagy* 9 609–611. 10.4161/auto.23460 23328265 PMC3627676

[B16] Jin Z. P. Pei Y. X. (2015). Physiological implications of hydrogen sulfide in plants: pleasant exploration behind its unpleasant odour. *Oxid. Med. Cell Longev.* 2015:397502. 10.1155/2015/397502 26078806 PMC4442293

[B17] Jin Z. P. Pei Y. X. (2016). Hydrogen sulfide: the shutter button of stomata in plants. *Sci. China Life Sci.* 59 1187–1188. 10.1007/s11427-016-0265-3 27778221

[B18] Jin Z. P. Shen J. J. Qiao Z. J. Yang G. D. Wang R. Pei Y. X. (2011). Hydrogen sulfide improves drought resistance in *Arabidopsis thaliana*. *Biochem. Biophys. Res. Commun.* 414 481–486. 10.1016/j.bbrc.2011.09.090 21986537

[B19] Jin Z. P. Wang Z. Q. Ma Q. X. Sun L. M. Zhang L. P. Liu Z. Q. (2017). Hydrogen sulfide mediates ion fluxes inducing stomatal closure in response to drought stress in *Arabidopsis thaliana*. *Plant Soil* 419 141–152. 10.1007/s11104-017-3335-5

[B20] Jin Z. P. Xue S. W. Luo Y. N. Tian B. H. Fang H. H. Li H. (2013). Hydrogen sulfide interacting with abscisic acid in stomatal regulation responses to drought stress in Arabidopsis. *Plant Physiol. Biochem.* 62 41–46. 10.1016/j.plaphy.2012.10.017 23178483

[B21] Kadoya F. Kato S. Watanabe K. Kato-Yamada Y. (2011). ATP binding to the ϵ subunit of thermophilic ATP synthase is crucial for efficient coupling of ATPase and H^+^ pump activities. *Biochem. J.* 437 135–140. 10.1042/BJ20110443 21510843

[B22] Kato Y. Matsui T. Tanaka N. Muneyuki E. Hisabori T. Yoshida M. (1997). Thermophilic F_1_-ATPase is activated without dissociation of an endogenous inhibitor, ε subunit. *J. Biol. Chem.* 272 24906–24912. 10.1074/jbc.272.40.24906 9312092

[B23] Kim C. Y. Bove J. Assmann S. M. (2008). Overexpression of wound-responsive RNA-binding proteins induces leaf senescence and hypersensitive-like cell death. *New Phytol.* 180 57–70. 10.1111/j.1469-8137.2008.02557.x 18705666

[B24] Li Z. G. Gong M. Xie H. Yang L. Li J. (2012). Hydrogen sulfide donor sodium hydrosulfide-induced heat tolerance in tobacco (*Nicotiana tabacum* L.) suspension cultured cells and involvement of Ca^2+^ and calmodulin. *Plant Sci.* 18 185–189. 10.1016/j.plantsci.2011.10.006 22325880

[B25] Lowry O. H. Rosebrough N. J. Farr A. L. Randall R. J. (1951). Protein measurement with the folin phenol reagent. *J. Biol. Chem.* 193 265–275. 14907713

[B26] Lyons J. Wheaton T. Pratt H. (1964). Relationship between the physical nature of mitochondrial membranes and chilling sensitivity in plants. *Plant Physiol.* 39 262–268. 10.1104/pp.39.2.262 16655908 PMC550065

[B27] Meyer T. Burow M. Bauer M. Papenbrock J. (2003). Arabidopsis sulfurtransferases: investigation of their function during senescence and in cyanide detoxification. *Planta* 217 1–10. 10.1007/s00425-002-0964-5 12721843

[B28] Munné-Bosch S. Alegre L. (2004). Leaf senescence contributes to plant survival under drought stress. *Funct. Plant Biol.* 31 203–216. 10.1071/FP03236 32688892

[B29] Niitepõld K. Hanski I. (2013). A long life in the fast lane: positive association between peak metabolic rate and lifespan in a butterfly. *J. Exp. Bot.* 216 1388–1397. 10.1242/jeb.080739 23264490

[B30] Noctor G. Paepe R. D. Foyer C. H. (2007). Mitochondrial redox biology and homeostasis in plants. *Trends Plant Sci.* 12 125–134. 10.1016/j.tplants.2007.01.005 17293156

[B31] Papenbrock J. Riemenschneider A. Kamp A. Schulz-Vogt H. N. Schmidt A. (2007). Characterization of cysteine-degrading and H_2_S-releasing enzymes of higher plants—from the field to the test tube and back. *Plant Biol.* 9 582–588. 10.1055/s-2007-965424 17853358

[B32] Rödiger A. Baudisch B. Klösgen R. B. (2010). Simultaneous isolation of intact mitochondria and chloroplasts from a single pulping of plant tissue. *J. Plant Physiol.* 167 620–624. 10.1016/j.jplph.2009.11.013 20045215

[B33] Rondelez Y. Tresset G. Nakashima T. Kato-Yamada Y. Fujita H. Takeuchi S. (2005). Highly coupled ATP synthesis by F_1_-ATPase single molecules. *Nature* 433 773–777. 10.1038/nature03277 15716957

[B34] Sõti C. Sreedhar A. S. Csermely P. (2003). Apoptosis, necrosis and cellular senescence: chaperone occupancy as a potential switch. *Aging Cell* 2 39–45. 10.1046/j.1474-9728.2003.00031.x 12882333

[B35] Toribarn J. R. Chen P. S. Warner H. (1966). Assay of inorganic phosphate, total phosphate and phosphatases. *Method Enzymol.* 8 115–118.

[B36] Wang L. X. Ma X. Y. Che Y. M. Hou L. X. Liu X. Zhang W. (2015). Extracellular ATP mediates H_2_S-regulated stomatal movements and guard cell K^+^ current in a H_2_O_2_-dependent manner in Arabidopsis. *Sci. Bull.* 60 419–427. 10.1007/s11434-014-0659-x

[B37] Wang R. (2012). Physiological implications of hydrogen sulfide: a whiff exploration that blossomed. *Physiol. Rev.* 92 791–896. 10.1152/physrev.00017.2011 22535897

[B38] Wang W. G. Yang X. L. Silanes I. L. Carling D. Gorospe M. (2003). Increased AMP: ATP ratio and AMP-activated protein kinase activity during celluar senescence linked to reduced HuR function. *J. Bio. Chem.* 278 27016–27023. 12730239 10.1074/jbc.M300318200

[B39] Yang G. D. Zhao K. X. Ju Y. J. Mani S. Cao Q. H. Puukila S. (2013). Hydrogen sulfide protects against cellular senescence *via S*-sulfhydration of Keap1 and activation of Nrf2. *Antioxid. Redox Sign.* 18 1906–1919. 10.1089/ars.2012.4645 23176571

[B40] Zhang H. Hu L. Y. Hu K. D. He Y. D. Wang S. H. Luo J. P. (2008). Hydrogen sulfide promotes wheat seed germination and alleviates oxidative damage against copper stress. *J. Integr. Plant Biol.* 50 1518–1529. 10.1111/j.1744-7909.2008.00769.x 19093970

[B41] Zhang H. Hu S. L. Zhang Z. J. Hu L. Y. Jiang C. X. Wei Z. J. (2011). Hydrogen sulfide acts as a regulator of flower senescence in plants. *Postharvest Biol. Technol.* 60 251–257. 10.1016/j.postharvbio.2011.01.006

[B42] Zhang H. Tang J. Liu X. P. Wang Y. Yu W. Peng W. Y. (2009). Hydrogen sulfide promotes root organogenesis in *Ipomoea batatas*, *Salix matsudana* and *Glycine max*. *J. Integr. Plant Biol.* 51 1086–1094. 10.1111/j.1744-7909.2009.00885.x 20021556

