# Supplementary material for: Hydrogen Sulfide Regulates Energy Production to Delay Leaf Senescence Induced by Drought Stress in Arabidopsis
Source: Front Plant Sci. 2018 Nov 23;9:1722. doi: 10.3389/fpls.2018.01722 (PMC6265512; doi:10.3389/fpls.2018.01722)
Supplement: Supplementary file 1 [file Presentation_1.pptx]

## Slide 1
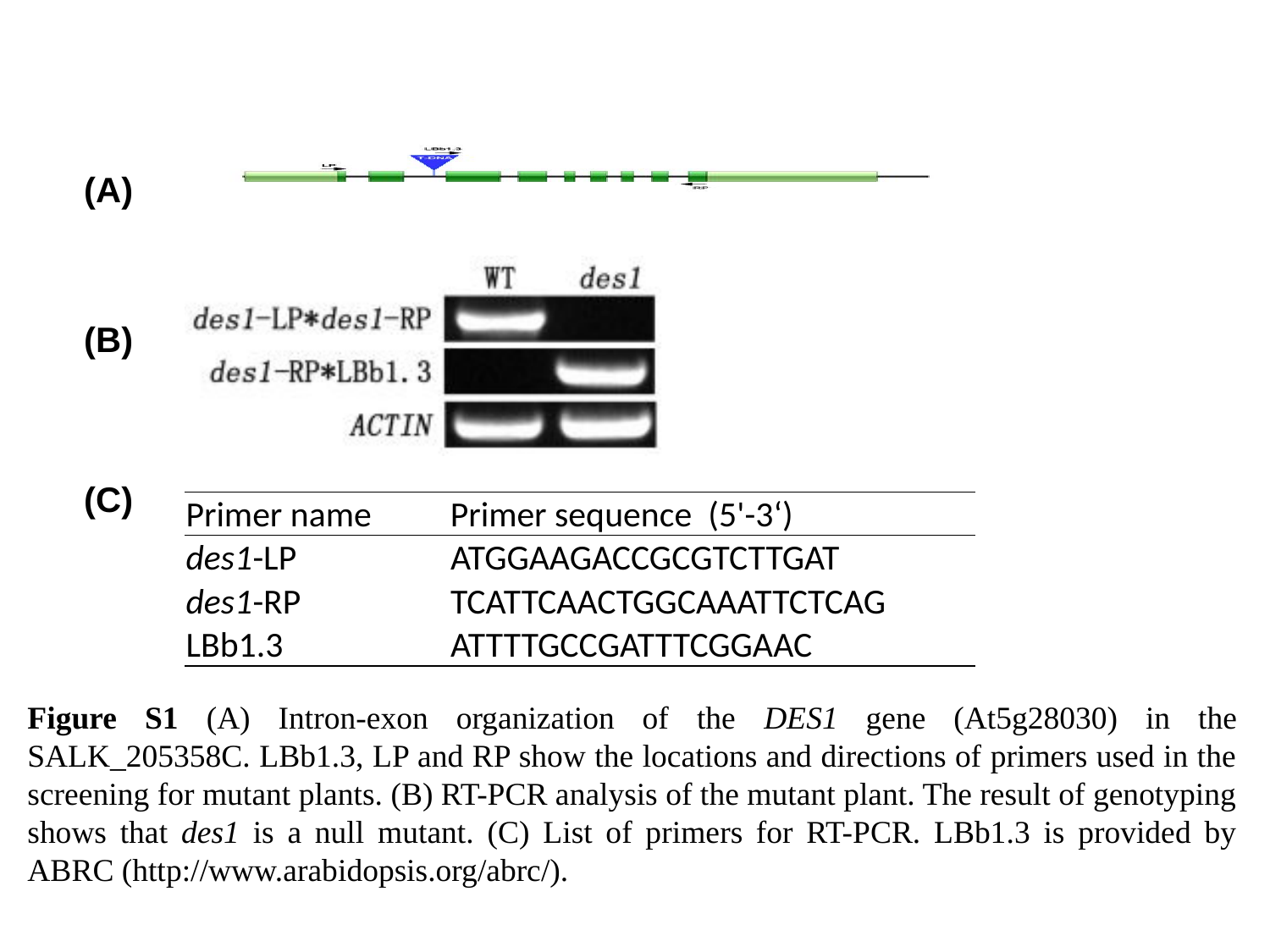

(A)
(B)
(C)
| Primer name | Primer sequence (5'-3‘) |
| --- | --- |
| des1-LP | ATGGAAGACCGCGTCTTGAT |
| des1-RP | TCATTCAACTGGCAAATTCTCAG |
| LBb1.3 | ATTTTGCCGATTTCGGAAC |
Figure S1 (A) Intron-exon organization of the DES1 gene (At5g28030) in the SALK_205358C. LBb1.3, LP and RP show the locations and directions of primers used in the screening for mutant plants. (B) RT-PCR analysis of the mutant plant. The result of genotyping shows that des1 is a null mutant. (C) List of primers for RT-PCR. LBb1.3 is provided by ABRC (http://www.arabidopsis.org/abrc/).

## Slide 2
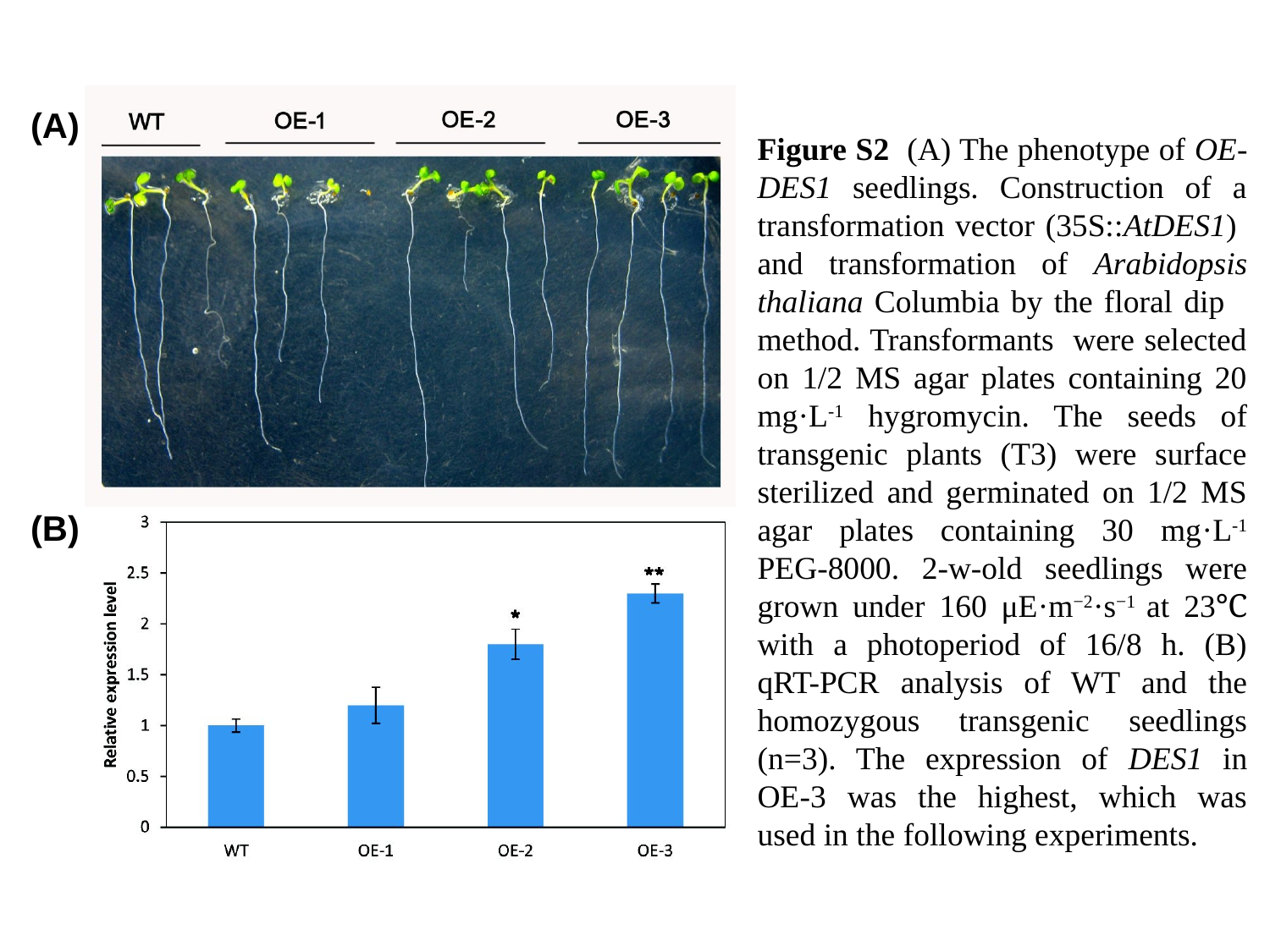

(A)
(B)
Figure S2 (A) The phenotype of OE-DES1 seedlings. Construction of a transformation vector (35S::AtDES1) and transformation of Arabidopsis thaliana Columbia by the floral dip method. Transformants were selected on 1/2 MS agar plates containing 20 mg·L-1 hygromycin. The seeds of transgenic plants (T3) were surface sterilized and germinated on 1/2 MS agar plates containing 30 mg·L-1 PEG-8000. 2-w-old seedlings were grown under 160 μE·m−2·s−1 at 23℃ with a photoperiod of 16/8 h. (B) qRT-PCR analysis of WT and the homozygous transgenic seedlings (n=3). The expression of DES1 in OE-3 was the highest, which was used in the following experiments.

## Slide 3
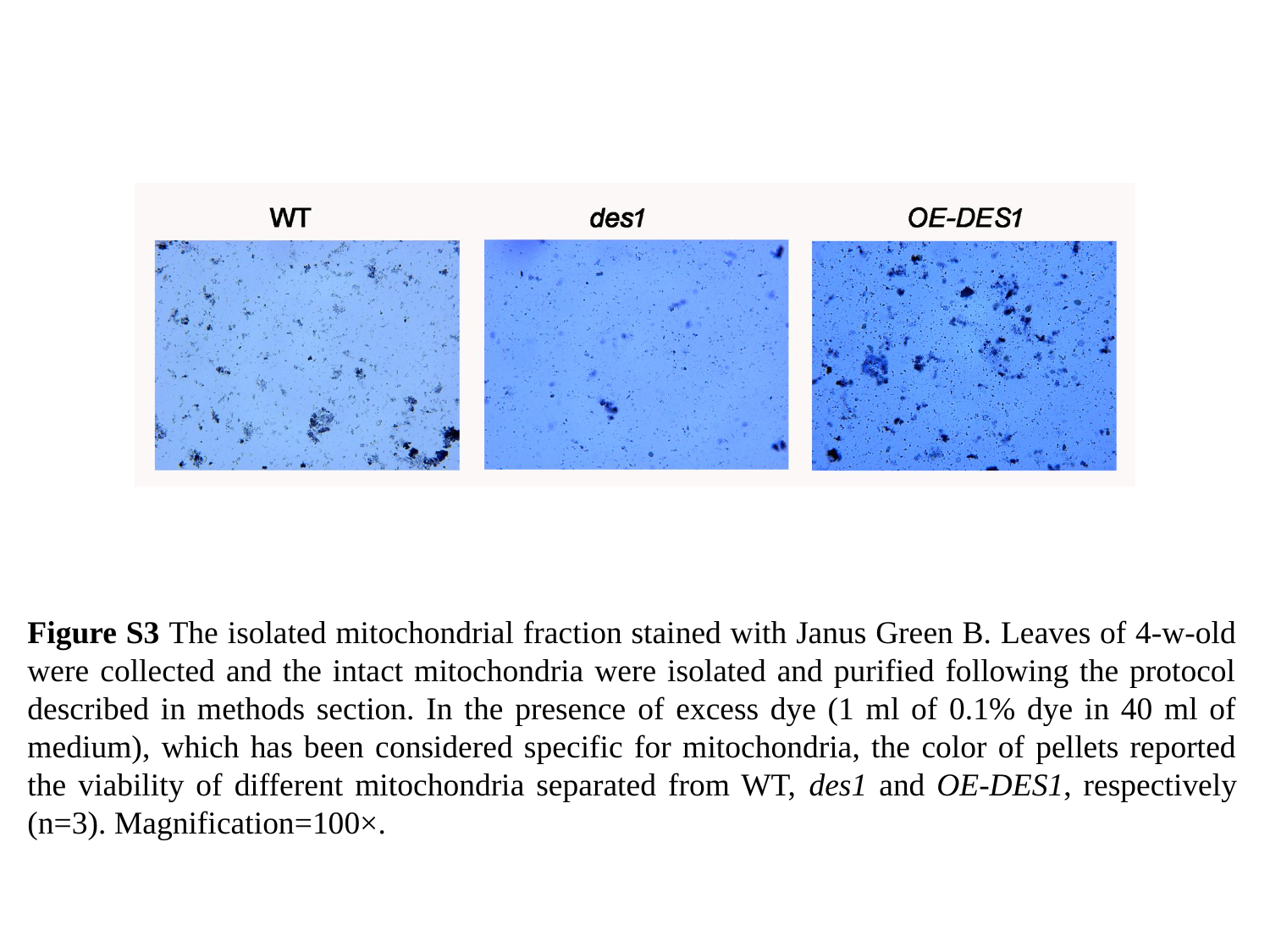

Figure S3 The isolated mitochondrial fraction stained with Janus Green B. Leaves of 4-w-old were collected and the intact mitochondria were isolated and purified following the protocol described in methods section. In the presence of excess dye (1 ml of 0.1% dye in 40 ml of medium), which has been considered specific for mitochondria, the color of pellets reported the viability of different mitochondria separated from WT, des1 and OE-DES1, respectively (n=3). Magnification=100×.

## Slide 4
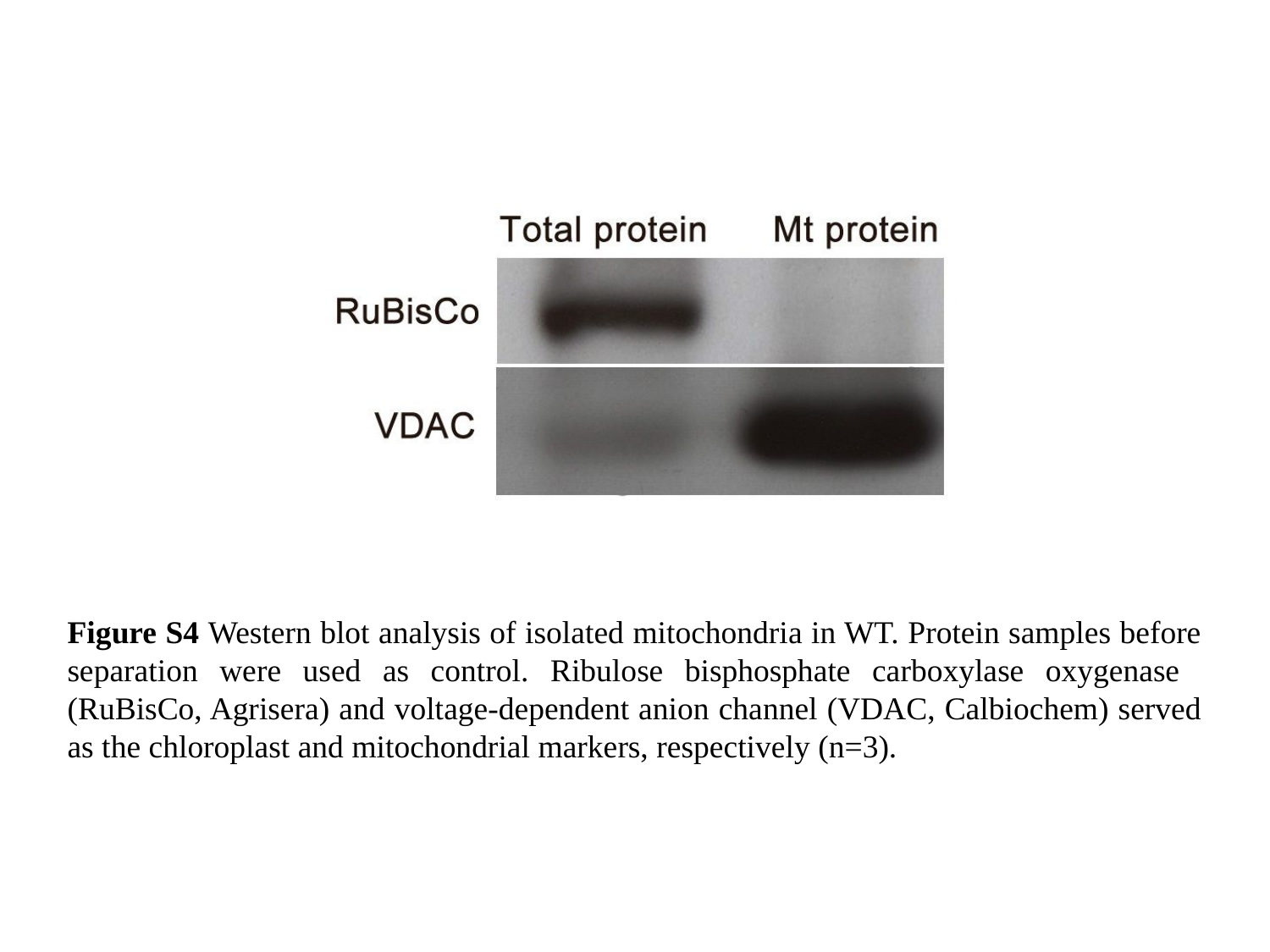

Figure S4 Western blot analysis of isolated mitochondria in WT. Protein samples before separation were used as control. Ribulose bisphosphate carboxylase oxygenase (RuBisCo, Agrisera) and voltage-dependent anion channel (VDAC, Calbiochem) served as the chloroplast and mitochondrial markers, respectively (n=3).

## Slide 5
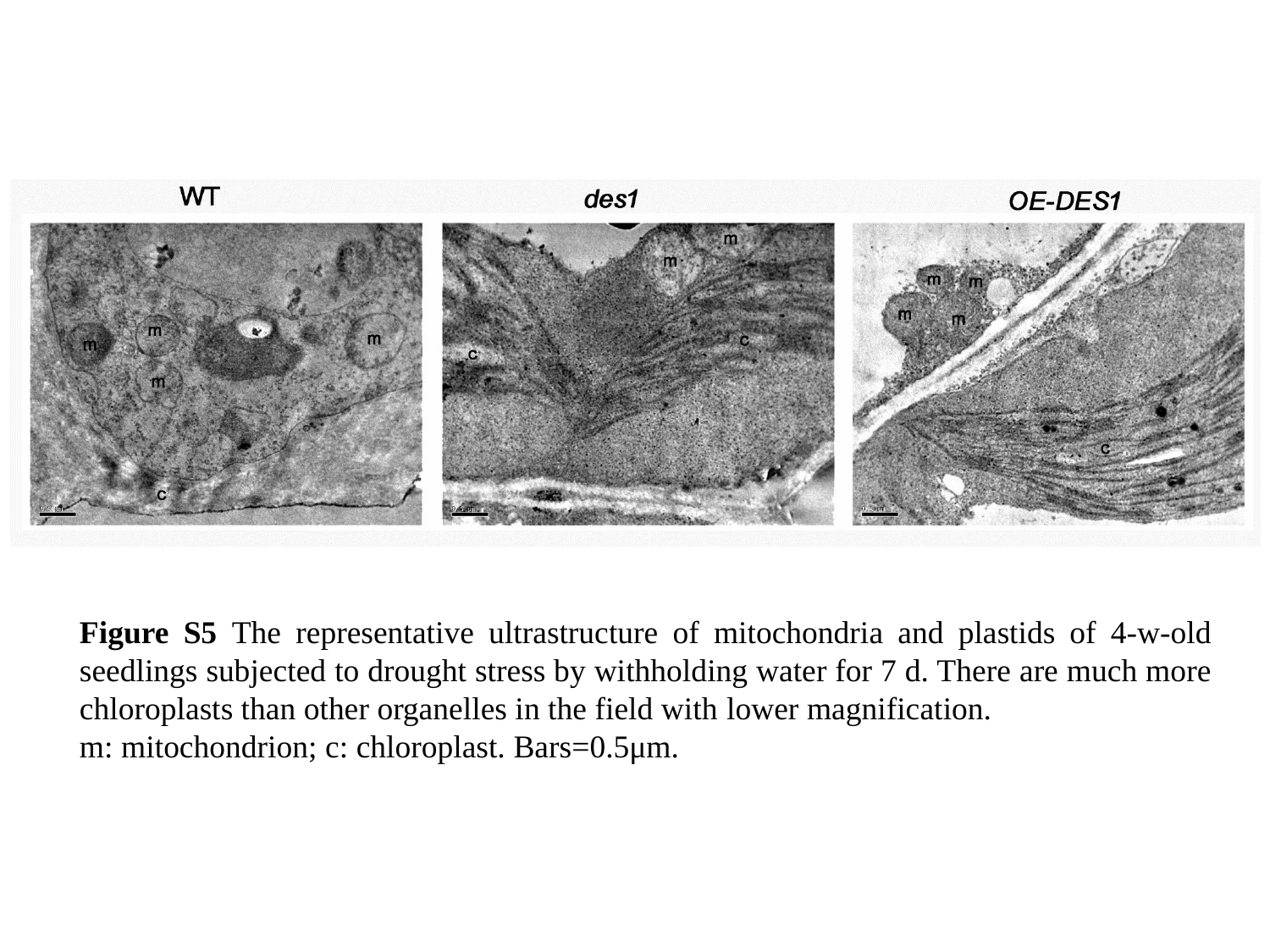

Figure S5 The representative ultrastructure of mitochondria and plastids of 4-w-old seedlings subjected to drought stress by withholding water for 7 d. There are much more chloroplasts than other organelles in the field with lower magnification.
m: mitochondrion; c: chloroplast. Bars=0.5μm.

## Slide 6
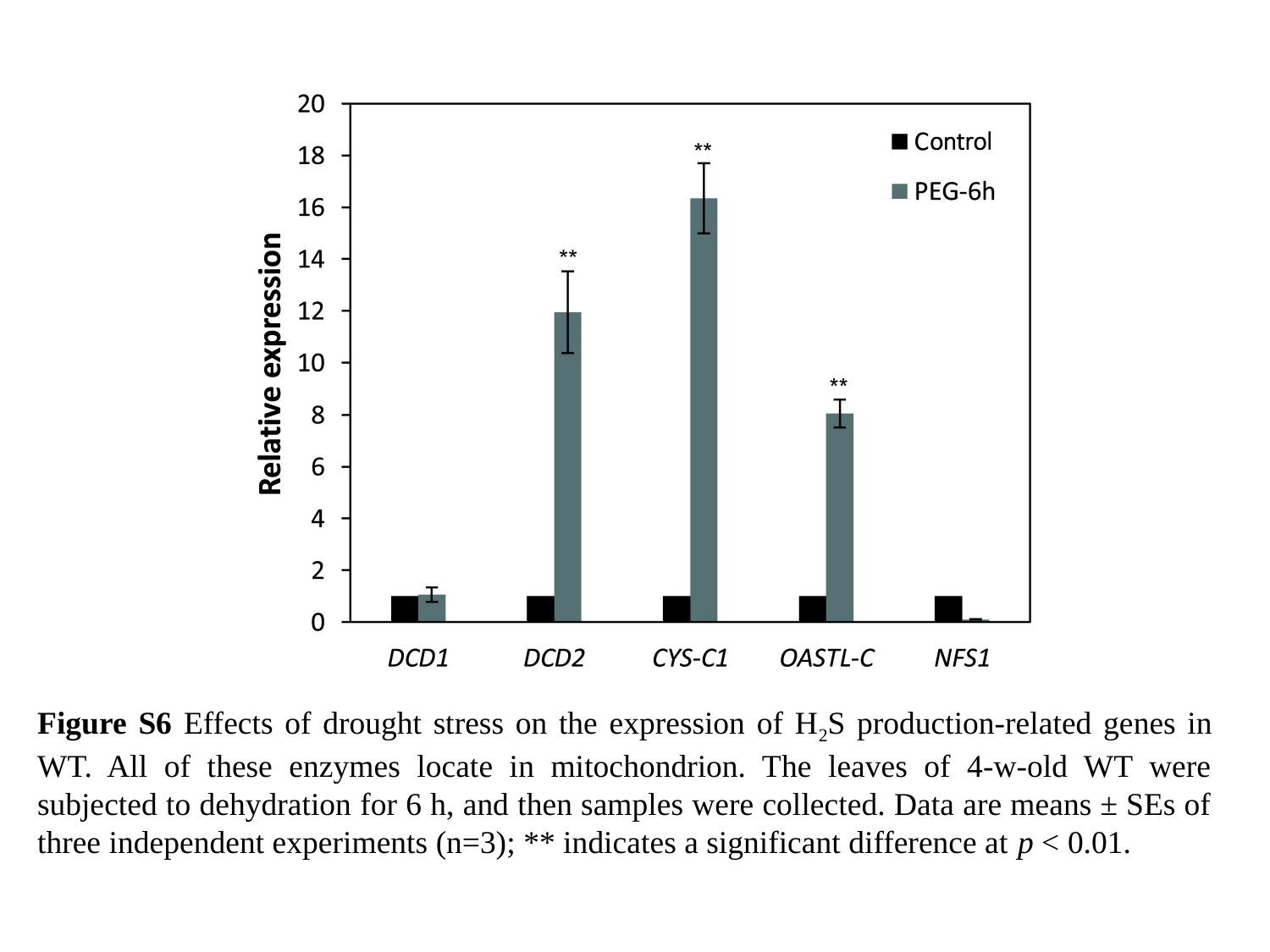

Figure S6 Effects of drought stress on the expression of H2S production-related genes in WT. All of these enzymes locate in mitochondrion. The leaves of 4-w-old WT were subjected to dehydration for 6 h, and then samples were collected. Data are means ± SEs of three independent experiments (n=3); ** indicates a significant difference at p < 0.01.
